# Delineating proinflammatory microenvironmental signals by ex vivo modeling of the immature intestinal stroma

**DOI:** 10.1038/s41598-021-86675-4

**Published:** 2021-03-30

**Authors:** Mari Ichinose, Nobumi Suzuki, Tongtong Wang, Josephine A. Wright, Tamsin R. M. Lannagan, Laura Vrbanac, Hiroki Kobayashi, Krystyna Gieniec, Jia Q. Ng, Souzaburo Ihara, Chris Mavrangelos, Yoku Hayakawa, Patrick Hughes, Daniel L. Worthley, Susan L. Woods

**Affiliations:** 1 School of Medicine, University of Adelaide, Adelaide, SA 5000 Australia; 2 South Australian Health and Medical Research Institute, Adelaide, SA 5000 Australia; 3 Department of Gastroenterology, Graduate School of Medicine, University of Tokyo, Tokyo, Japan

**Keywords:** Infant necrotizing enterocolitis, Inflammation

## Abstract

The intestinal stroma provides an important microenvironment for immune cell activation. The perturbation of this tightly regulated process can lead to excessive inflammation. We know that upregulated Toll-like receptor 4 (TLR4) in the intestinal epithelium plays a key role in the inflammatory condition of preterm infants, such as necrotizing enterocolitis (NEC). However, the surrounding stromal contribution to excessive inflammation in the pre-term setting awaits careful dissection. Ex vivo co-culture of embryonic day 14.5 (E14.5) or adult murine intestinal stromal cells with exogenous monocytes was undertaken. We also performed mRNAseq analysis of embryonic and adult stromal cells treated with vehicle control or lipopolysaccharide (LPS), followed by pathway and network analyses of differentially regulated transcripts. Cell characteristics were compared using flow cytometry and pHrodo red phagocytic stain, candidate gene analysis was performed via siRNA knockdown and gene expression measured by qPCR and ELISA. Embryonic stromal cells promote the differentiation of co-cultured monocytes to CD11b^high^CD11c^high^ mononuclear phagocytes, that in turn express decreased levels of CD103. Global mRNAseq analysis of stromal cells following LPS stimulation identified TLR signaling components as the most differentially expressed transcripts in the immature compared to adult setting. We show that CD14 expressed by CD11b^+^CD45^+^ embryonic stromal cells is a key inducer of TLR mediated inflammatory cytokine production and phagocytic activity of monocyte derived cells. We utilise transcriptomic analyses and functional ex vivo modelling to improve our understanding of unique molecular cues provided by the immature intestinal stroma.

## Introduction

Excessive gastrointestinal tract (GIT) inflammation can be problematic. This is seen with potentially lethal effect in the most frequent GIT disease in preterm infants, necrotizing enterocolitis (NEC). NEC patients present with an abnormal gut microbiota^1^ and disease typified by excessive GIT inflammation. Toll-like receptor 4 (TLR4), a receptor for lipopolysaccharide (LPS) produced by Gram-negative bacteria, is upregulated in the intestinal epithelium in NEC and plays a key role in the etiology of this disease^2,3^. Cluster of differentiation 14 (CD14) is a co-receptor for Toll Like Receptors (TLRs), and sensitizes cells to LPS by transferring bacterial cell wall products to TLR4^4,5^. The homodimerization of TLR4 is prompted by key interactions with myeloid differentiation factor 2 (MD-2), and triggers downstream myeloid differentiation primary response gene 88 (MyD88) and TIR-domain-containing adapter-inducing interferonβ (TRIF) dependent production of pro-inflammatory cytokines and type I interferons^5,6^. TRIF-dependent signaling is linked with endocytosis of the activated TLR4, which is controlled by CD14. Increased TLR4, CD14 and MyD88 have previously been reported in human NEC intestinal samples, and the inhibition of these molecules with an anti-CD14 antibody, systemic mutation of TLR4, or systemic knock out of MyD88, significantly attenuates disease severity in NEC rodent models^2,7,8^. However, the cell-type specific localization of TLR4, MyD88, and CD14 in the tissue and interplay between different cellular components of the tissue microenvironment leading to excessive inflammation in the immature intestine remains to be fully elucidated.

An inflammation associated immune imbalance exaggerates tissue damage in preterm infants^1,3^. Studies using tissue isolated from infants with NEC or experimental mouse models have implicated intestinal macrophages in the pathogenesis of NEC^9^. Mononuclear phagocytes (MPs), comprising macrophages and dendritic cells (DCs), have a key role in discriminating between harmful and harmless antigens in the lumen of the gut. Intestinal MPs engulf damaged tissue, whereas regulatory MPs help to repair and regenerate the intestinal epithelium. Macrophages and immature DCs continuously sample foreign antigens in peripheral tissues via endocytosis. After capturing antigens, DCs migrate to lymphoid tissues where they present antigen to T cells^10^. We previously reported that CD11c^+^ MPs directly interact with the intestinal epithelium, inhibit goblet cell differentiation, and determine subsequent inflammatory responses^11,12^.

The bone marrow stroma provides factors essential for the maturation of macrophage/dendritic cell precursors (MDPs). Monocytes arising from MDPs are released to the circulating blood. Monocytes then differentiate into various MP lineage, depending on local microenvironmental cues^13,14^. The localized signals that shape monocyte differentiation and activation in the intestinal mucosa have recently attracted attention^14–16^. The intestinal stroma consists of several cell types including myofibroblasts, fibroblasts, pericytes, bone marrow-derived mesenchymal stem cells, smooth muscle cells of the muscularis mucosae, and immune cells^17^. This complex cell mix provides secreted growth factors and ligands which regulate intestinal homeostasis^18–20^. How these microenvironmental cues differ between the embryonic and adult states is yet to be fully investigated, but may be key to furthering our understanding of immune mis-regulation. We hypothesize that the immature intestinal stroma may lack the appropriate cues found in the adult to generate regulatory MPs to assist with repair of the epithelial barrier. To test this hypothesis, we assessed the effect of primary embryonic stromal cells on monocyte differentiation ex vivo. Use of an ex vivo co-culture system allows us to model this monocyte modulatory effect and mechanistically explore subsequent potential inflammatory responses in the immature intestine. We also performed mRNAseq analysis using primary stromal cells collected from embryonic and adult mouse small intestines to identify transcripts that were differentially expressed between the immature and adult setting. Using gene set enrichment and network analyses we identified *CD14* as a hub gene induced in the embryonic stroma in response to inflammatory stimuli.

## Materials and methods

### Mouse

Mouse lines were on the C57BL6/J background and experimentation was conducted following approval by SAHMRI Animal Ethics Committee (approval number SAM272) in accordance with the Australian code for the care and use of animals for scientific purposes and complying with the ARRIVE guidelines, with no blinding undertaken. UBC-GFP mice (C57BL/6-Tg (UBC-GFP) 30Scha/J, JAX 004353) were purchased from Jackson laboratory.

### Intestinal stromal cell culture

Intestinal stromal cells were collected from the mouse small intestine as described previously^21^ with some modifications. Adult mouse small intestines (SI) were removed, opened longitudinally, and villi mechanically scraped off. The much smaller embryonic mouse SI was collected using a dissection microscope and cut into 2 mm pieces. The intestines were incubated with chelation buffer (3 mM EDTA, 0.05 mM DTT (Sigma) in PBS) for 30 min at room temperature. The epithelial layer was removed with shaking in PBS. The remaining intestines were digested using 3.8 mg/ml Collagenase XI (Sigma) and 0.1 mg/ml Dispase II (Roche) for 15 min at 37 °C. The resulting supernatant was passed through a 70 µm strainer and single cells were pelleted by centrifugation at 300 *g* for 5 min and plated in stromal cell media: RPMI1640 supplemented with 5% FBS, 2 mM L-glutamine (GIBCO), 10 mM HEPES, 1 mM sodium pyruvate, 100 U/ml penicillin and 100 mg/ml streptomycin (GIBCO), 50 µM β-mercaptoethanol, and 1 µM Y27632 until confluent. Cells were used at passage 2–3. Lipopolysaccharides (LPS) from Eschericia coli O111:B4 (Sigma) at a final concentration of 10 µg/µl was added to the culture medium and cultured at 37 °C in 5%CO_2_ for 6 h. For co-culture with bone marrow cells, the intestinal stromal cells were seeded at a density of 1*10^6 per well in a 10 cm dish and cultured at 37 °C in 5%CO_2_ for 2 days until the cells reached 80% confluency. Bone marrow cells freshly collected from the tibias of UBC-GFP mice were added to the stromal cells at a density of 8*10^6 per well in a 10 cm dish, and co-cultured for a further 2 days. For co-culture with monocytes, the intestinal stromal cells were seeded at the density of 1.7*10^5 per well in a 6 well plate and cultured at 37 °C in 5%CO_2_ for 2 days until the cells reached 80% confluency. Monocytes, collected as stated below, were added to the stromal cells at the density of 4*10^5 per well in a 6 well plate, and co-cultured for a further 2 days. Bone marrow cells or monocytes were co-cultured with stromal cells in stromal cell media but with 10% FBS.

### Monocyte isolation

Monocytes were isolated from the bone marrow of UBC-GFP mice by negative selection using a MACS cell-separation system with a monocyte isolation kit, according to the manufacturer’s instructions (Miltenyi Biotec, catalogue # 130-100-629). CD3^-^B220^-^NK1.1^-^CD11b^+^Ly6G^-^Ly6C^+^ monocytes were collected at a purity of 95.9% (Suppl Fig. 1), as determined by flow cytometry.

### Flow cytometry

Dissociated cells were prepared and stained with DAPI (Sigma, 0.5 µg/ml) or Fixable viability stain 575 V (BD 56594) to enable the elimination of dead cells. Cells were stained with CD11b (BD 557657), CD11c (BioLegend 117328), CD103 (BD 564322), CD14 (BioRad MCA2745PE), CD45 (BioLegend 103108), CD3 (BD 563565), B220 (eBioscience 12045281), NK1.1 (BD 564144), Ly6G (BioLegend 127609), Ly6C (BioLegend 128037) and CD326 (Epcam) (BioLegend 118205). GFP was used to identify bone marrow cells originating from UBC-GFP donor mice. Data was acquired on an LSR Fortessa X-20 cell analyzer (Becton–Dickinson, San Jose, CA) and analyses were carried out using FlowJo software (Tree Star).

To analyse the purity of monocytes isolated using MACS cell separation, dead cells and red blood cells were excluded with FSC, SSC and FVS575V. CD3^-^B220^-^ gated non T/B cells were further analyzed for NK1.1 and CD11b expression. NK1.1^-^CD11b^+^ cells were then analyzed further for Ly6G and Ly6C expression to detect Ly6G^-^Ly6C^+^ monocytes (Suppl Fig. 1).

**Figure1:**
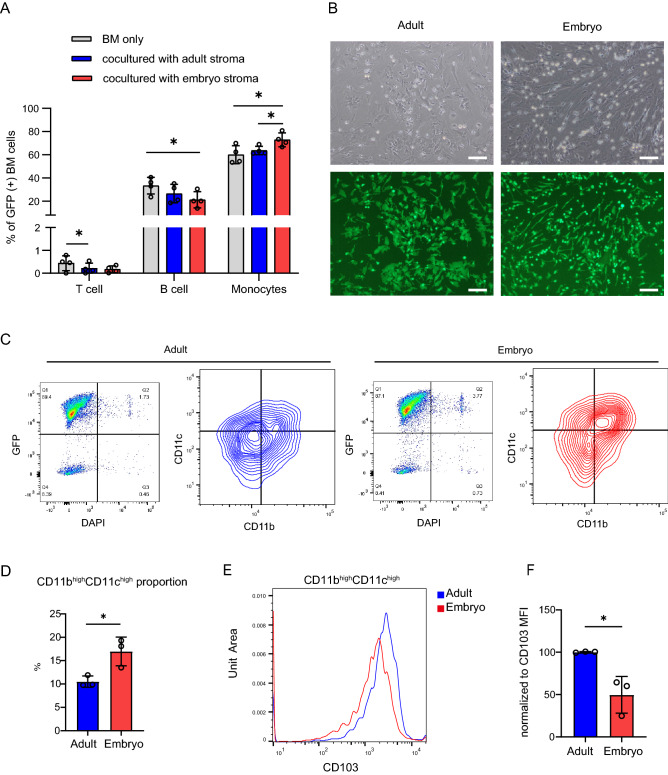
Co-culture with embryonic intestinal stroma promotes differentiation of exogenous monocytes into CD11b^high^CD11c^high^ CD103^low^ MPs. (**A**) Mouse GFP + bone marrow cells were co-cultured ex vivo with wild type adult or embryonic intestinal stroma for 2 days. The differentiation state of live GFP + cells was analyzed by flow cytometry. T cells; CD3^+^, B cells; B220^+^, myelomonocytic cells; CD3^-^B220^-^CD11b^+^ n = 4 biological replicates. One way ANOVA with Tukey’s multiple test. *p < 0.05. (**B**) Representative bright field and fluorescent images of GFP labelled monocyte derived cells co-cultured with wild type intestinal adult or embryonic stromal cells for 2 days. Scale bar = 100 µm. (**C**) DAPI^-^ GFP^+^ live monocyte derived cells from ex vivo coculture of stromal cells and monocytes shown in (**B**), were analyzed for CD11b and CD11c expression by flow cytometry. (**D**) Quantification of (**C**) showing the percentage of CD11b^high^CD11c^high^ cells. n = 3 biological replicates. t-test. *p < 0.05. (**E**) Histogram showing CD103 expression in CD11b^high^CD11c^high^ gated cells from (**C**). (**F**) CD103 MFI in CD11b^high^CD11c^high^ gated cells from (**C**) normalized to adult stroma. n = 3 biological replicates. t-test. *p < 0.05.

To determine whether CD14 was expressed on intestinal stromal cells, DAPI^-^ live cells were analysed for CD45 and CD11b expression. Then, CD14 expression on CD45^-^CD11b^-^ and CD45^+^CD11b^+^ populations was examined. To measure the expression of CD3, B220, and CD11b in GFP labelled bone marrow cells co-cultured with wild-type stromal cells, DAPI^-^ GFP^+^ live cells were analyzed for CD3 and B220 expression. Then, CD11b expression was analyzed on CD3^-^B220^-^ gated cells (Suppl Fig. 2). To examine CD11b, CD11c, and CD103 expression on GFP labelled monocytes co-cultured with wild-type stromal cells, DAPI^-^ GFP^+^ live cells were analyzed for CD11b and CD11c expression. CD103 expression was analyzed in the CD11b^high^CD11c^high^ gated population (Fig. 1C).

### Phagocytosis assay

Adult or embryonic intestinal stromal cells were cultured in a 96 well plate and treated with LPS at 10 µg/ml for 15 h. The cells were washed 3 times with PBS and GFP labelled monocytes were then added to the culture for 2 days, after which the cells were incubated with pHrodo Red E. Coli BioParticles at the final concentration of 50 µg/ml (Thermo Fisher Scientific) for 2 h. Once engulfed by phagocytic cells, pH-induced red fluorescence was visualized and images were acquired using an Evos FL Auto2 Imaging System (Thermo Fisher Scientific).

### RNA extraction and mRNAseq analysis

RNeasy mini kits (Qiagen) were used to isolate RNA from cultured cells. RNA quality and quantity were analyzed using a NanoDrop and TapeStation. PolyA + stranded RNA-seq libraries were prepared using the KAPA RNA Hyper Prep kit (Roche). The multiplexed libraries were sequenced on an Illumina NextSeq 500 to obtain a single-end 75 bp read. The fastq files from sequencing run were firstly subject to quality controls using FastQC version 0.11.3. Raw reads with low quality (Phred score less than 28, reads contains adaptor sequences) were removed using Trim Galore. The trimmed reads were mapped to NCBI mouse genome (GRCm38.p6) with STAR 2.4.2a^22^. No more than 1 base mismatch were allowed and only uniquely mapped reads were retained. Over 97% of reads mapped to the genome for each sequenced sample. The GEO accession number for all sequencing data reported in this paper is GSE161707.

### Differential expression analysis and functional annotations

**Figure 2:**
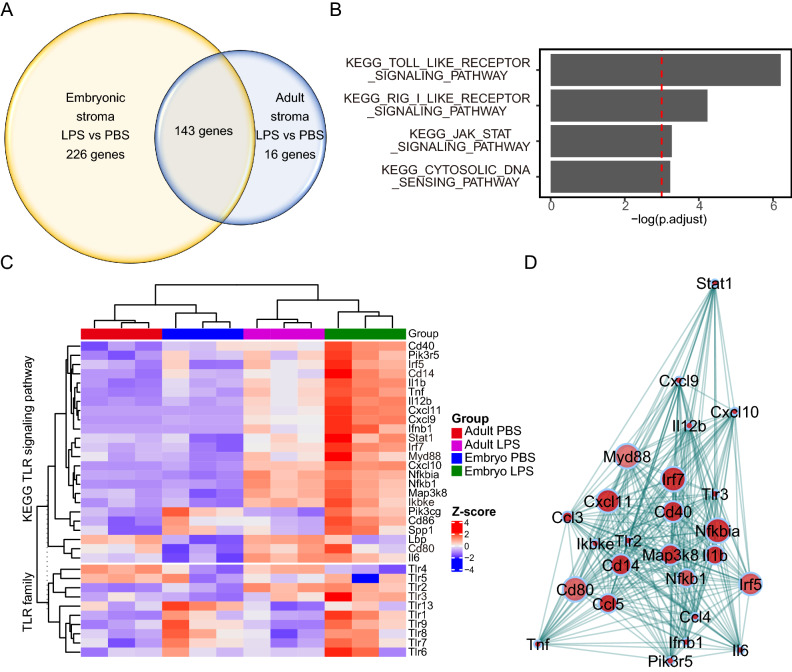
Identification of key genes differentially expressed by the immature mouse intestinal stroma upon inflammatory stimulation ex vivo. (**A**) Venn diagram showing the number of differentially expressed genes (DEGs) in primary adult or embryonic intestinal stromal cells treated with LPS in comparison to vehicle controls. 3 independent cultures generated from 3 adult mice or 6 pooled embryos/culture were sequenced. DEG had absolute value of log2 fold change ≥ 2.0, FDR < 0.05. (**B**) Significantly enriched KEGG pathways for 226 genes differentially expressed in LPS stimulated embryonic stromal cells. FDR < 0.05. (**C**) Heatmap generated using ComplexHeatmap R package version 2.1.0^27^ depicting unsupervised clustering of TLR and TLR pathway component expression in LPS or control vehicle treated embryonic and adult stromal cells ex vivo. (**D**) Network visualization constructed using R package WGCNA^28^ of the correlation between DEG in LPS stimulated embryonic stromal cells in the KEGG TLR signaling pathway. Each dot represents a gene, dot interior colour denotes upregulation (red) of gene transcript in LPS treated cells compared to PBS treated cells, dot size indicates the magnitude of gene expression correlation to neighboring genes, lines connecting dots represent topological distance.

TMM normalized counts were log2-transformed to obtain counts per million (CPM). Only expressed genes were retained for further analysis (defined as cpm > 0.5 for all samples in a group). Differential gene expression analysis was then conducted using the edgeR package in R version 3.6.0^23^ to contrast transcripts expressed in embryonic stromal cells treated with LPS compared to vehicle control (PBS) and adult stromal cells treated with LPS compared to PBS. We selected genes significantly differentially expressed by LPS stimulation in the embryonic setting only using cutoff of logFC > 2 and FDR < 0.01 (226 genes) and performed hypergeometric enrichment tests with clusterProfiler R package version 3.13.0^24^, using the KEGG gene sets in the Molecular Signature Database version 6.2^25,26^. Bonferroni adjustment for p value was used, with only gene sets with an adjusted p value less than 0.05 considered (Fig. 2B). To visualize components of the most significant, enriched pathway, differentially expressed genes were inspected using ComplexHeatmap R package version 2.1.0^27^ (Fig. 2C).

### Supervised weighted gene correlation network analysis (WGCNA)

To identify key molecules within the toll like receptor signaling pathway that may contribute to the activation of inflammation in the embryonic stroma, we performed network analysis. Gene networks were constructed using the R package WGCNA following the procedure described by Langfelder and Horvath^28^. In brief, a pairwise Pearson’s correlation based adjacency matrix was calculated for DEGs from embryonic intestinal stromal cells treated with LPS in comparison to vehicle, found in the toll like receptor signaling pathway. The topological overlap of the correlation was used to weight the edges of the correlation network. The higher the weight, the stronger the implied interaction between expression of two genes (network plot weight cut-off was 0.6). The connectivity for a single gene was calculated as the sum of the weights for that gene in relation to the rest of the genes, and nodes within the top 10th percentile for connectivity in the network were defined as hub genes.

### Real time RT-PCR

RNA was isolated using an RNeasy mini kit (Qiagen) and reverse-transcribed into cDNA with cDNA master (Sigma). PCR amplification was performed with Kappa SYBR qPCR mix using QuantStudio7 (Applied Biosystems). The mouse GAPDH gene was used as an endogenous control to normalize across samples. The following primers were used:

Tlr4-forward, GCATGGCTTACACCACCTCT.

Tlr4-reverse, TTTGTCTCCACAGCCACCAG.

Il1b-forward, TGCCACCTTTTGACAGTGATG.

Il1b-reverse, TGATGTGCTGCTGCGAGATT.

Tnf-forward, CATCTTCTCAAAATTCGAGTGACAA.

Tnf-reverse, TGGGAGTAGACAAGGTACAACCC.

Cd14-forward, ACTGAAGCCTTTCTCGGAGC.

CD14-reverse ,TGAAAGCGCTGGACCAATCT.

### siRNA

Invitrogen Silencer Select siRNA (Carlsbad, CA) targeting *Cd14* at a final concentration of 5 µM was utilized for gene silencing using Lipofectamine RNAiMAX reagent (Invitrogen), following the manufacturers’ instructions. Cells were harvested 48 h after transfection.

### Enzyme-linked immunosorbent assay (ELISA)

Adult or embryonic stromal cell supernatants were concentrated tenfold with Amicon Ultra 100 K (UFC9100, Merck). TNF-α and IL1β levels in the culture medium of control and LPS treated stromal cells were determined using commercially available ELISA kits that were purchased from BD Biosciences and Invitrogen, respectively, in accordance with the manufacturer’s instructions.

### Statistics

All statistical analyses were performed using Graphpad prism 8. Values are presented as mean ± SD. The data was analyzed by one-way ANOVA, followed by Tukey’s post hoc multiple-comparisons test or unpaired t-test as indicated in the figure legends. The significance level was set at P < 0.05.

## Results

### The embryonic intestinal stroma enhances monocyte differentiation to CD11b^high^CD11c^high^ MPs

To investigate whether there are different microenvironmental cues provided by the embryonic and adult stroma that influence incoming immune cell behavior, we developed an ex vivo co-culture system. This contained primary mouse GFP-labelled bone marrow cells to model exogenous blood cell behavior and wild-type intestinal embryonic or adult stromal cells. Cells were co-cultured for 2 days and then GFP^+^ cells analysed by flow cytometry for immune cell differentiation markers. This included a particular focus on MP cells given their key role in discriminating harmful and harmless stimuli. The proportion of CD11b^+^ CD3^-^ B220^-^ myelomonocytic cells in the GFP^+^ population significantly increased when co-cultured with embryonic stromal cells, at the expense of B220^+^ B cells, and to a lesser extent, CD3^+^ T cells. This effect was much dampened following co-culture with adult stroma compared with the embryonic stroma (Fig. 1A).

CD11b is a marker of monocytes as well as a subset of NK cells and granulocytes. To focus our studies on the monocytes that give rise to MPs, CD3^-^B220^-^NK1.1^-^CD11b^+^Ly6G^-^Ly6C^+^ monocytes were enriched from GFP labelled bone marrow cells using a MACS monocyte isolation kit (Suppl Fig. 1). Similar to our previous experiment, the enriched monocyte population was co-cultured with embryonic or adult intestinal stromal cells for 2 days (Fig. 1B). Morphological analysis indicated that monocyte derived GFP^+^ cells had a bi-polar, thin, elongated shape following co-culture with embryonic stroma while co-culture with adult stroma generated cell populations with broader cell bodies with multipolar elongations. To characterize the immunophenotype of these GFP labelled monocyte derived cells we used flow cytometry to analyse expression of the leukocyte markers, CD11b, CD11c, and CD103 in GFP^+^ cells. A higher proportion of CD11b^high^CD11C^high^ monocytes were observed following co-culture with embryonic stroma than with adult stroma (Fig. 1C,D). Interestingly, CD103 expression was significantly upregulated in CD11b^high^CD11C^high^GFP^+^ cells co-cultured with adult stroma compared to embryonic stroma (Fig. 1E,F). CD103^+^ DCs induce the differentiation of Foxp3^+^ regulatory T cells from naïve T cells^29^, thus the decrease of CD103 on MPs in the embryonic stroma may result in the reduction of Tregs and subsequent immune activation.

### The immature intestinal stroma displays a heightened transcriptional response to inflammatory stimulation ex vivo

To understand at the molecular level why preterm infants may have a propensity to develop excessive GIT inflammation, we asked what the differences were in embryonic and adult stromal responses to an inflammatory stimulus. For this we used LPS, a component of the outer wall of Gram-negative bacteria that is commonly used to model the inflammatory response to bacterial exposure, such as occurs after birth. We performed a global mRNAseq analysis of primary intestinal embryonic and adult stromal cells challenged with LPS or vehicle controls. Multidimensional scaling plot analysis grouped biological replicates together but presented clear separation of sample transcriptomes based on developmental stage and also LPS stimulation (Suppl. Figure 3). 143 genes were differentially regulated by LPS in both the embryonic and adult stroma in comparison to the respective vehicle treated controls (Fig. 2A). These included genes known to be regulated by LPS such as Nuclear Factor Kappa B Subunit 1 (*Nfkb1*), interleukin 1β (*Il1b*) and tumor necrosis factorα (*Tnf*). Somewhat unexpectedly, we also identified 226 uniquely differentially expressed genes (DEGs) following LPS stimulation of embryonic stromal cells compared to vehicle control, and only 16 unique DEG after LPS treatment of adult stromal cells compared to vehicle controls (Fig. 2A). This suggests a broader transcriptional reprogramming occurs in embryonic compared to adult stroma following LPS exposure. Pathway analysis of the 226 uniquely DEG in embryonic stroma indicated that components of the Toll-like receptor signaling pathway were most enriched (Fig. 2B). The Retinoic acid-inducible gene I (RIG I) like receptor signaling pathway, Janus kinase-signal transducer and activator of transcription (JAK STAT) signaling pathway, and cytosolic DNA sensing pathway were also significantly enriched (Fig. 2B). The RIG I like receptor signaling pathway contains pattern recognition receptors for double-stranded RNA viruses^30^. We focused our further investigations on the most enriched gene set, containing members of the Toll-like receptor signaling pathway. Heat map visualization of transcripts encoding TLR signaling components and the TLR family (Fig. 2C) showed that unexpectedly, Toll-like receptor 4 (*Tlr4*) itself was expressed at lower levels in embryonic stromal cells compared to adult. This finding was confirmed by qPCR (Suppl Fig. 4A). In contrast, in the epithelial compartment, *Tlr4* mRNA expression was higher in embryonic compared to adult intestinal cells by qPCR, consistent with previous reports describing high neonatal levels of *Tlr4/TLR4* expression in the intestinal epithelium^2^ (Suppl Fig. 4B).

To determine key nodes of the TLR signaling pathway that are altered by LPS in the embryonic stroma we undertook network analysis using our mRNAseq data. This network visualization displays how closely correlated the expression of different members of the TLR signaling pathway are in embryonic stromal cells upon LPS stimulation and identified hub genes, whose expression most closely correlated with other genes in the TLR pathway, shown in Fig. 2D. Among the hub genes, interferon regulatory factor5/7 (*Irf5/7*)*, Cd14, Stat1, Myd88,* Inhibitor of nuclear factor kappa-B kinase subunit epsilon (*Ikbke*)*, Tlr3,* and cluster of differentiation 80 (*Cd80*) were significantly upregulated in embryonic and not adult stroma upon stimulation. In order to most potently impede inflammation driven by the embryonic stroma, we considered the availability of blocking reagents targeting hub genes for potential future clinical use. As such we further focused on *Cd14* in this study given that CD14 blocking agents are in clinical trial for infectious diseases^31^.

### CD45^+^CD11b^+^ leukocytes in the embryonic stroma respond to LPS-stimulation by upregulating CD14

**Figure3:**
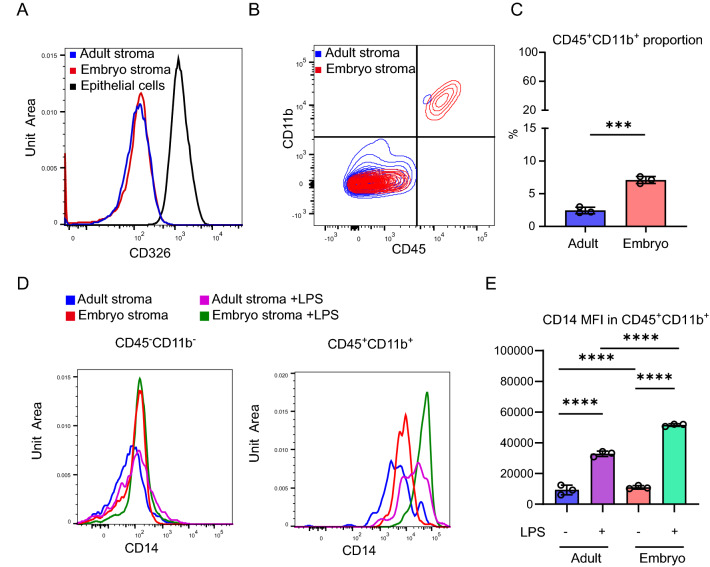
CD14 is upregulated in CD45^+^CD11b^+^ leukocytes in the embryonic stroma following LPS stimulation. (**A**) Histogram showing CD326 expression in primary adult or embryonic intestinal stromal cells, with intestinal epithelial cells as a positive control. Data representative of n = 3 independent cultures generated from 3 adult mice or 3 litters of 6 embryos each. (**B**,**C**) Flow cytometric analysis of CD45 and CD11b expression in adult or embryonic intestinal stromal cells. The percentage of live CD45^+^CD11b^+^ cells were compared. n = 3 biological replicates. t-test ***p < 0.001. (**D**) Histogram depicting CD14 expression in CD45^-^CD11b^−^ or CD45^+^CD11b^+^ populations in adult or embryonic intestinal stromal cells treated with LPS or vehicle control. (**E**) CD14 mean fluorescence intensity (MFI) in CD45^+^CD11b^+^ gated adult or embryonic intestinal stromal cells treated with LPS or vehicle. n = 3 biological replicates. One way ANOVA with Tukey’s multiple test. ****p < 0.0001.

The intestinal stroma is a heterogeneous collection of cells distinct from the epithelia. To identify the major source of CD14 in LPS stimulated embryonic stroma, we used flow cytometry to isolate distinct cell populations. As expected, our ex vivo embryonic and adult stromal cultures do not express CD326, in contrast to the positive control CD326^+^ intestinal epithelial cells by flow cytometry (Fig. 3A). We sorted CD326^-^, live stromal populations for immune cells using CD45 (pan-leukocyte marker) and CD11b (restricted to monocytes, macrophages, NK cells and granulocytes). Interestingly, the percentage of CD45^+^CD11b^+^ cells was significantly higher in the embryonic compared to adult stromal cultures (mean ± SD, adult: 2.45 ± 0.50%, embryo: 7.1 ± 0.53%) (Fig. 3B,C). Following LPS stimulation, CD14 expression was significantly increased on CD45^+^ CD11b^+^ cells in both the embryonic and adult stroma, and to a significantly greater extent in the embryonic setting (Fig. 3D,E). In contrast CD14 expression was lower in the CD45^-^CD11b^-^ cells compared to CD45^+^CD11b^+^and was not altered by LPS treatment in the embryonic or adult stroma (Fig. 3D,E). Taken together, these data suggested that the increased proportion of CD45^+^CD11b^+^ cells in the embryonic stroma upregulate CD14 expression following LPS stimulation, providing a differential microenvironmental cue between the embryonic and adult intestinal stroma.

### CD14 regulates inflammatory cytokine production and phagocytic behaviour of monocyte derived cells

**Figure 4:**
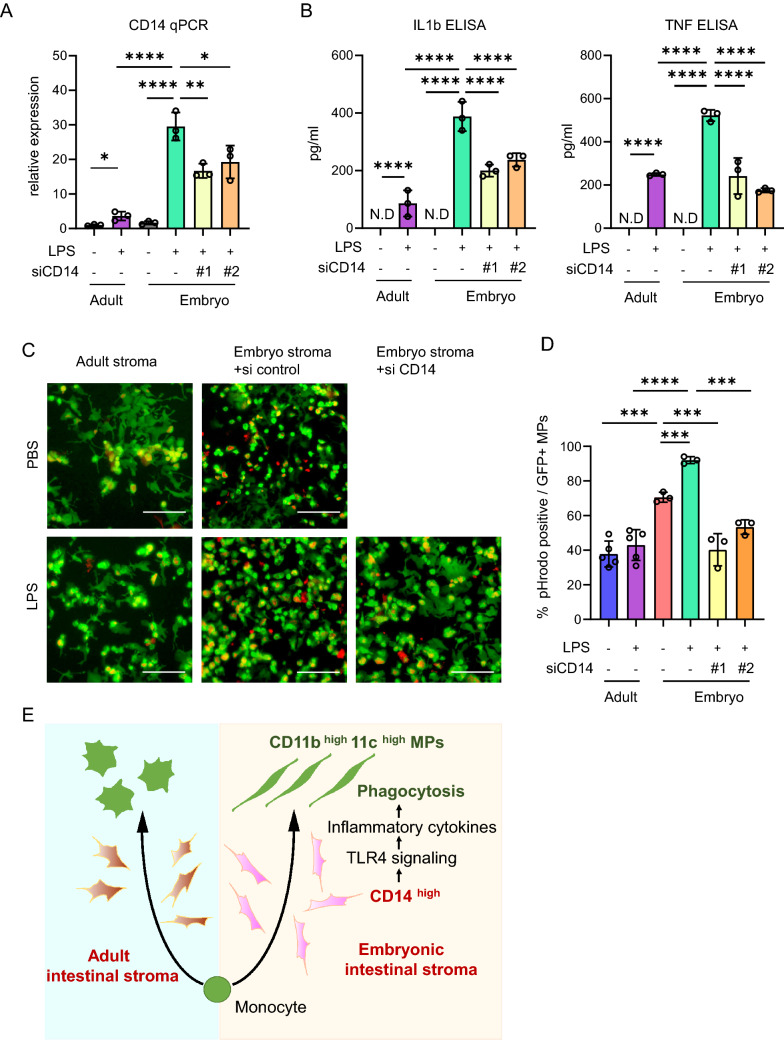
Inflammation induced CD14 in the embryonic intestinal stroma promotes inflammatory cytokine production and phagocytosis of monocyte derived cells. (**A**) *CD14* mRNA expression normalized to *Gapdh* in LPS or vehicle treated adult and embryonic intestinal stromal cells, with the addition of control or *CD14* siRNA knockdown in embryonic stromal cell cultures. n = 3 biological replicates. t-test. *p < 0.05, **p < 0.01, ****p < 0.0001. (**B**) IL1b and TNF ELISA using culture medium from LPS or vehicle treated adult and embryonic intestinal stromal cells, with the addition of control or *CD14* siRNA knockdown in embryonic stromal cell cultures. n = 3 biological replicates. t-test. ****p < 0.0001 N.D.; not detected. (**C**) Representative fluorescent images of phagocytic cells identified with pHrodo red fluorescence in co-cultures of adult or embryonic stromal cells and MACS sorted GFP + monocytes. Scale bar = 100 µm. (**D**) Quantification of (**C**) showing the percentage of pHrodo red/GFP^+^ out of the total GFP^+^ cells in 2 HPF of 3 biological replicates. n = 3 biological replicates. t-test., ***p < 0.001, ****p < 0.0001. (**E**) Graphical abstract of the findings.

To investigate the role of CD14 in stromally-driven inflammatory responses, we modified CD14 expression in our ex vivo stromal culture. As previously shown at the protein level (Fig. 3D,E), LPS treatment significantly increased *Cd14* transcript levels in both adult and embryonic stromal cells ex vivo, but with a significantly greater increase in the embryonic setting than in the adult (Fig. 4A). Knock down of *Cd14* with two independent siRNAs in comparison to scrambled control siRNA was validated by qPCR in embryonic stromal cells treated with LPS (Fig. 4A). Next we measured the effect of modulating CD14 expression on production of the pro-inflammatory cytokines IL-1b and TNFa. CD14 knock down significantly reduced TNF and IL1b expression, both at the RNA level as determined by qPCR (Suppl Fig. 5) and protein level by ELISA using the culture medium from LPS or vehicle treated embryonic stromal cells (Fig. 4B). To further examine the role of CD14 in the regulation of MP behavior in the proinflammatory embryonic stroma, we analysed phagocytosis in GFP labelled monocytes co-cultured with adult stromal cells and control or CD14 siRNA treated embryonic stromal cells. Phagocytic activity of exogenous MPs was assessed using pHrodo Red dye by determining the ratio of dual positive phagocytic pHrodo red^+^ and GFP^+^ cells, from all GFP^+^ cells. LPS treatment of adult stroma ex vivo did not increase phagocytosis in co-cultured GFP^+^ monocytes (Fig. 4C,D). In contrast, phagocytic activity in GFP^+^ MPs co-cultured with embryonic stroma was significantly increased by LPS treatment. CD14 knock down significantly reduced phagocytosis, suggesting that phagocytic behavior of recruited monocyte-derived MPs is at least partially dependent on CD14 function in the embryonic stroma (Fig. 4C–E).

## Discussion

At present, an increased reactivity of the immature intestinal epithelium to bacterial colonization is thought to generate an excessive inflammatory immune activation and NEC. The intestinal stroma is known to provide and regulate the local microenvironment and interact both with epithelial cells and immune cells. We need to understand the tissue distribution and spatial interactions of key inflammatory regulators in the preterm intestine. Global differences in molecular cues provided by the immature stroma have yet to be investigated in detail. Our bulk RNAseq analysis of intestinal stromal cells identified unique transcriptional responses to LPS in the embryonic compared to mature setting (Fig. 2A). Despite expression of *Tlr4* itself not being altered by LPS stimulation in stromal cells, *Cd14*, *MyD88* and *Irf5/7* were upregulated, suggesting TLR pathway activation and pro-inflammatory consequences mediated at least in part by the stroma (Fig. 2D). We observe upregulation of CD14 at the protein level also in our primary mouse intestinal stromal cell culture (Fig. 3D,E). CD14 is present in a soluble form, as well as on the surface of myelomonocytic cells and non-immune cells such as fibroblasts and mesenchymal stem cells^4,32,33^. Thus upregulation of CD14 in the embryonic intestinal stroma can enhance TLR4 mediated inflammatory responses in a paracrine manner in adjacent cells.

In the embryonic intestine, tissue-resident macrophages are derived from mesenchymal stem cells in the yolk sac and fetal liver monocytes. These cells are replenished by bone marrow-derived Ly6C^high^ monocytes after birth^34,35^. Circulating CD14^+^CD45^+^ fibrocytes arise from monocyte precursors and undergo recruitment and activation in several inflammatory conditions, such as NEC, inflammatory bowel disease, and cancers. In pancreatic cancer, in which the majority of the tumor volume is fibrotic stroma rather than neoplastic cells, fibrocytes are concentrated in the stroma surrounding the tumor. It is thought that they secrete extra cellular matrix and recruit new fibrocytes, myofibroblasts, and stellate cells around them^36^. Fibrocytes are also increased in the plasma, intestinal mucosa, and submucosal layers of NEC patients^37^, however the contribution of stroma to fibrocyte accumulation has remained elusive. Our study has revealed that ex vivo cultured embryonic intestinal stroma contains more CD14^+^CD45^+^ cells than adult stroma and CD14 expression was also significantly increased upon LPS stimulation (Fig. 3A–E). In addition, the embryonic stroma induced differentiation of bone marrow cells to CD11b^+^ cells (Fig. 1A) and promoted monocyte differentiation to CD11b^high^CD11c^high^ cells (Figs. 1C,D, 4E). Knock-down of CD14 alone was not sufficient to impede this monocyte differentiation and so multiple factors found in the embryonic stroma, including CD14^+^CD45^+^ cells are likely to cooperatively form monocyte differentiation niches. As candidate niche factors we considered stromal colony-stimulating factor (CSF)-1,2 and transforming growth factor beta (TGFβ)-1,2 which have been reported to have roles in the differentiation of MPs^9,16,38^. However, CSF-1,2 and TGFβ-1,2 mRNA expression was not significantly different between embryonic and adult stroma in our ex vivo cultures (Suppl Fig. 6). Thus, further efforts are required to identify the embryonic stromal factors promoting the differentiation of monocytes to CD11b^high^CD11c^high^ cells.

In NEC, polarization toward increased proinflammatory CD3^+^CD4^+^IL-17^+^ T cells and reduced tolerogenic Foxp3^+^ Treg lymphocytes is observed^3^. However regulation of the key MPs in the immature stroma that give rise to these lineages has not been studied in detail. We have shown here that CD11b^high^CD11c^high^ cells emerged following co-culture with embryonic stroma and expressed lower levels of CD103 than after co-culture with adult stroma (Figs. 1E,F,4E). As CD103^+^ DCs can promote the conversion of naïve T cells to Foxp3^+^ regulatory T cells^29^, we hypothesise that a decrease in CD103 expression on MPs in the embryonic stroma may contribute to the reduction in Treg lymphocytes observed in NEC, however this needs to be confirmed by future in vivo studies.

To further understand the potential mechanisms of cellular damage in the premature intestine, we investigated the functional differences in MPs co-cultured with embryonic or adult stromal cells (Fig. 4A). LPS stimulated embryonic stroma induced the phagocytic activity of co-cultured MPs. This is likely due to high CD11c expression in the MPs (Fig. 1C,D), which plays a critical role in antigen uptake^39^. Knock-down of *Cd14* in co-cultured embryonic stroma reduced the phagocytosis in MP cells, most likely because of an indirect effect of suppression of inflammation (Fig. 4C–E), rather than a direct effect on the differentiation of MPs. Taken together, the immature stroma induces highly phagocytotic MPs in the intestinal microenvironment. The subsequent antigen uptake in these MPs is likely to lead to further immune activation.

Inherent limitations of this study include the inability of our ex vivo culture system to contain all the many components of the microenvironment such as epithelial cells, lymph nodes, or blood vessels and so does not examine interactions with these components. Our novel culture model is useful, however, to investigate the cross-talk between stromal and immune components, but these findings need to be further validated in vivo. To further examine the potential stromal contribution to NEC pathology in the rodent model, the developmental time-point examined should also be extended to encompass several developmental stages. Indeed, the developmental stage investigated here is slightly earlier than NEC rodent models in other studies^3,7^. Also, bacterial stimulation rather than the use of LPS will be useful to recapitulate and understand inflammatory settings in humans.

In summary, we have shown that the ex vivo culture of intestinal stromal cells collected from embryonic mice is a useful research tool to elucidate molecular features of the immature intestine. In the premature intestinal stroma, CD14 expressed on monocyte lineage cells plays pivotal roles in inflammation. These results support the validity of CD14 as a target therapy for NEC and help clarify its mechanism of action.

## Supplementary Information

Supplementary Information
