# Supplementary material for: Delineating proinflammatory microenvironmental signals by ex vivo modeling of the immature intestinal stroma
Source: Sci Rep. 2021 Mar 30;11:7200. doi: 10.1038/s41598-021-86675-4 (PMC8010037; doi:10.1038/s41598-021-86675-4)
Supplement: Supplementary file 1 — Supplementary Information [file 41598_2021_86675_MOESM1_ESM.pdf]

Supplementary Files- Delineating proinflammatory microenvironmental signals by ex vivo modeling of the immature intestinal stroma.

Mari Ichinose[1,2)*], Nobumi Suzuki[1,2,3)*], Tongtong Wang[1,2)], Josephine A Wright[1,2)], Tamsin R M Lannagan[1,2)], Laura Vrbanac[1,2)], Hiroki Kobayashi[1,2)], Krystyna Gieniec[1,2)], Jia Q Ng[1,2)], Souzaburo Ihara[3)], Chris Mavrangelos[1,2)], Yoku Hayakawa[3)], Patrick Hughes[1,2)], Daniel L Worthley[2)], Susan L Woods[1,2)]

1. School of Medicine, University of Adelaide, Adelaide, SA 5000, Australia

2. South Australian Health and Medical Research Institute, Adelaide, SA 5000, Australia

3. Department of Gastroenterology, Graduate School of Medicine, University of Tokyo, Tokyo, Japan

Ichinose et al., Suppl figure1

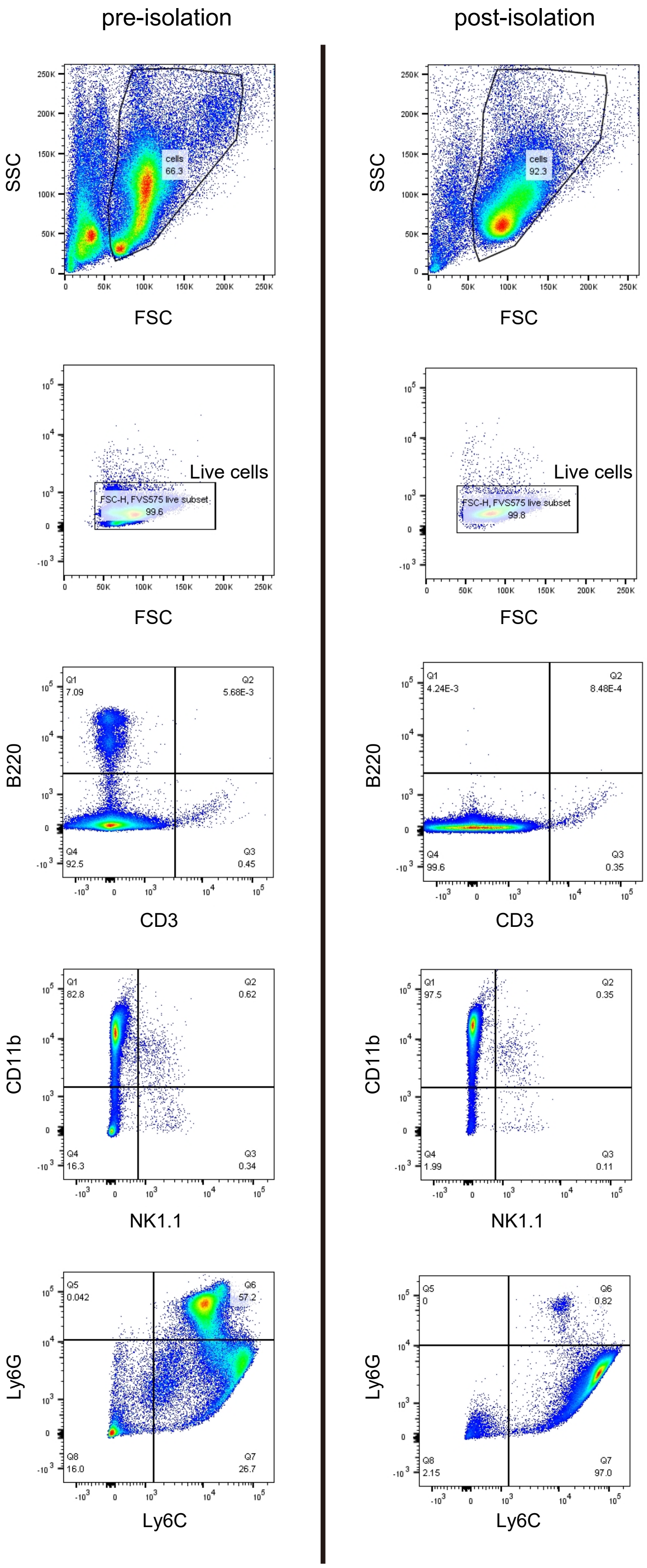

Suppl Fig.1 FACS validation of MACS enrichment of CD3-B220-NK1.1-CD11b+Ly6G-Ly6C+ monocytes from mouse bone marrow cells.

Ichinose et al., Suppl Figure2

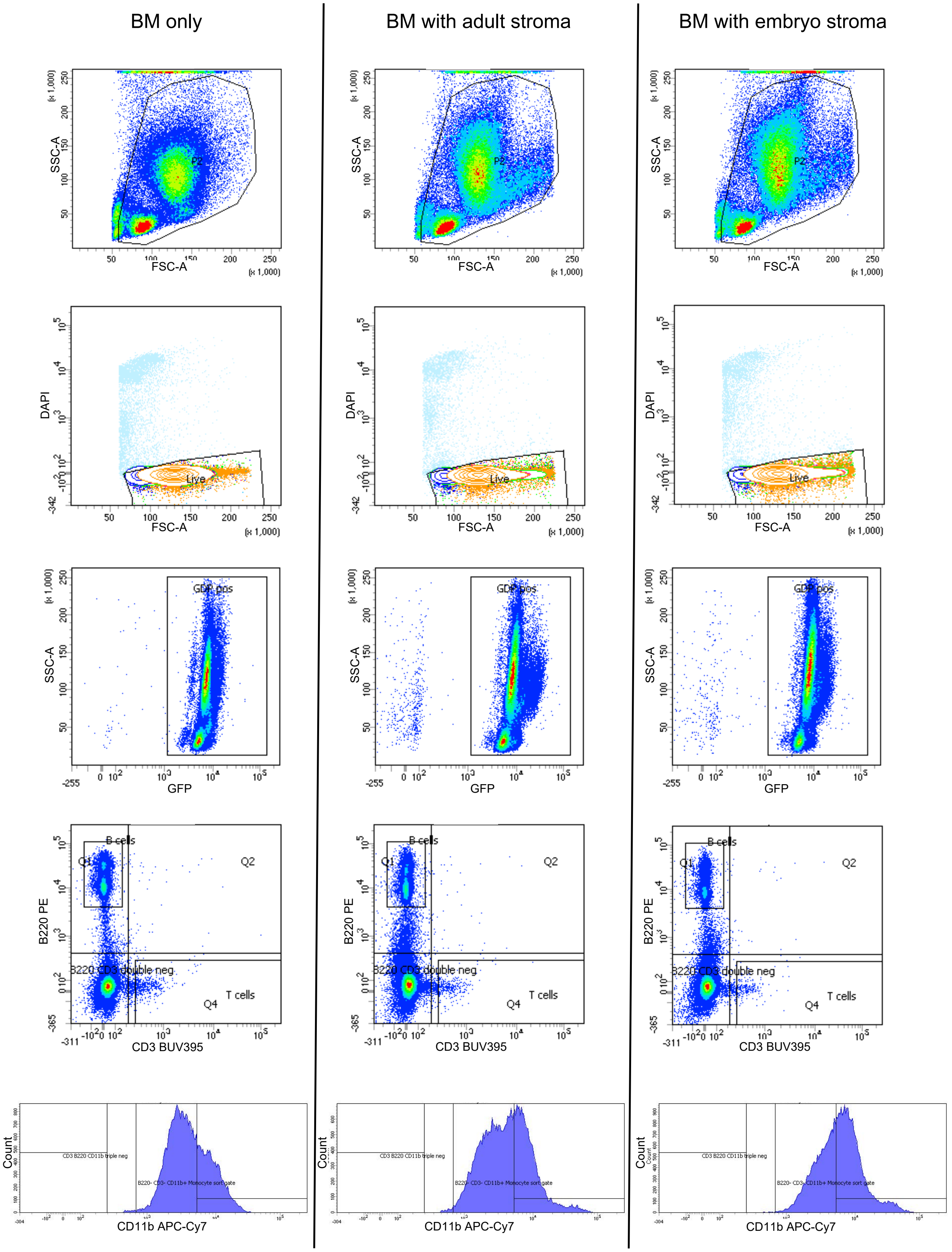

Suppl Fig.2 FACS gating strategy to identify the proportion of T cells, B cells, and myelomonocytic cells in co-cultures of wild-type intestinal stromal cells and GFP+ bone marrow derived cells.
DAPI- GFP+ live bone marrow cells were classified into CD3+ T cells, B220+ B cells, and CD3-B220-CD11b+ myelomonocytic cells. The proportion of each cell population is shown in Fig.1A.

Ichinose et al., Suppl figure3

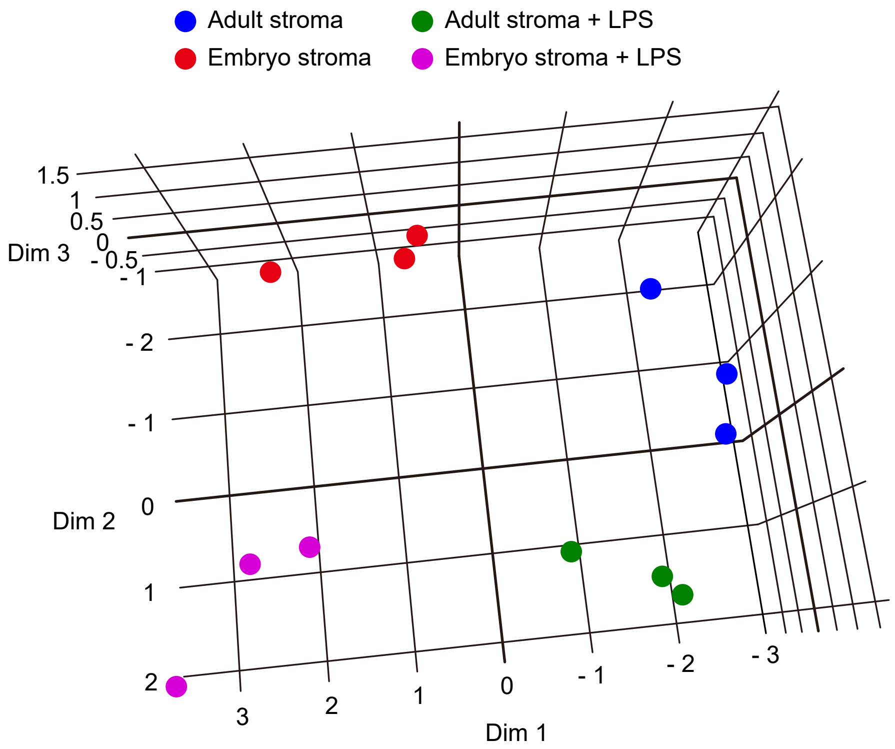

Suppl Fig.3 Multidimensional scaling plot comparison of transcriptome of LPS or control vehicle treated embryonic and adult stromal cells ex vivo.

Ichinose et al., Suppl Figure4

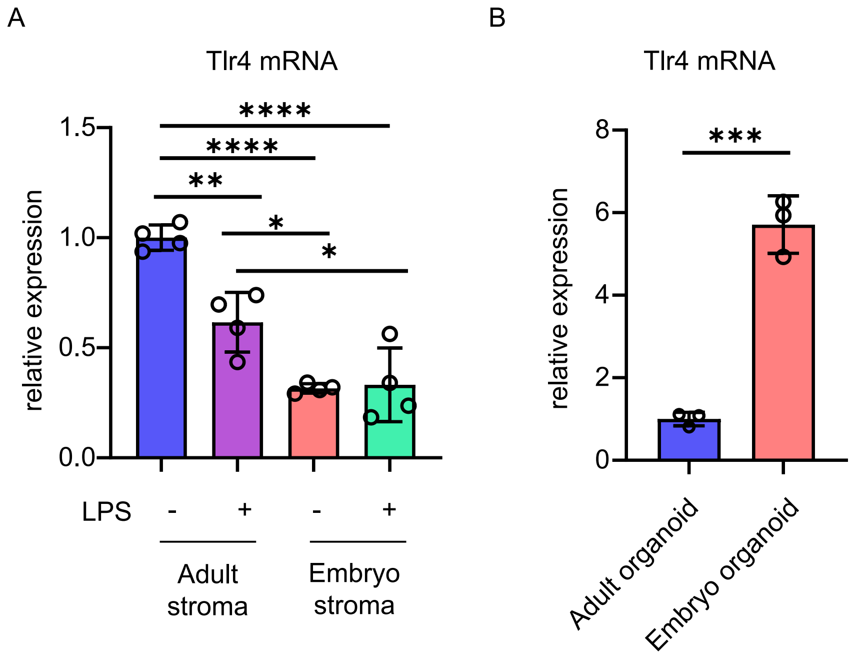

Suppl Fig.4 TLR4 expression is not induced in the embryonic or adult intestinal stroma following LPS stimulation, but is upregulated in embryonic intestinal epithelium in comparison to adult epithelium.
(A) TLR4 mRNA expression normalized to Gapdh in adult and embryonic stromal cells treated with LPS or vehicle by qPCR. N=4 biological replicates. t-test. *$p<0.05$, **$p<0.01$, ****$p<0.0001$
(B) TLR4 mRNA expression normalized to Gapdh in adult and embryonic small intestinal organoids (epithelial cells) by qPCR. N=3 biological replicates. t-test. ***$p<0.001$

Ichinose et al., Suppl figure5

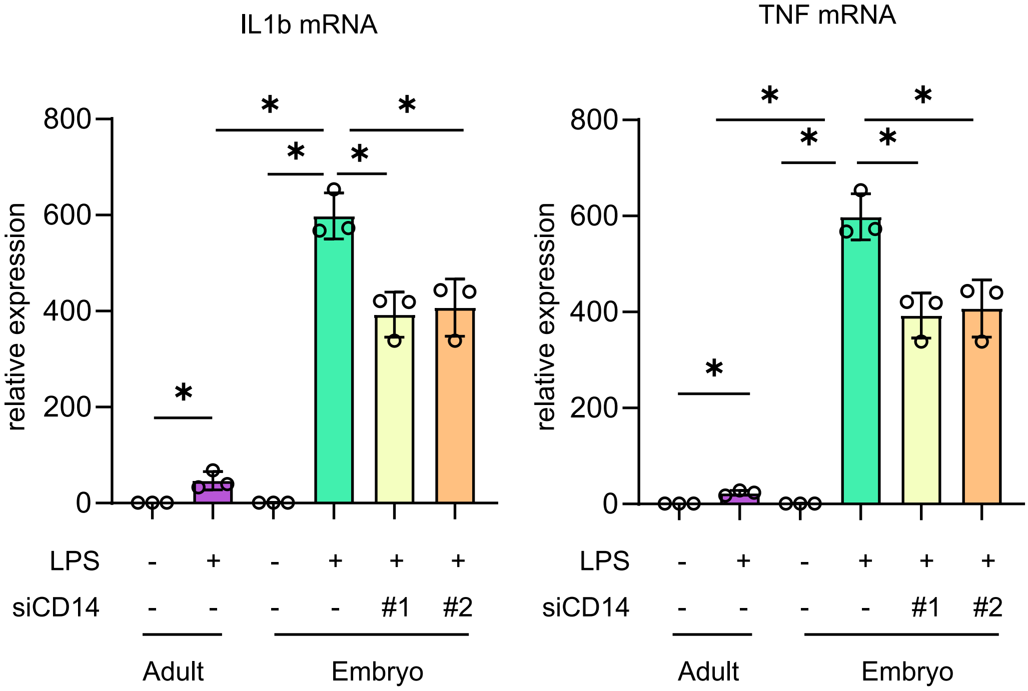

Suppl Fig.5 CD14 regulates inflammatory cytokine production from monocyte derived cells.
IL1b and TNF mRNA expression normalized to Gapdh in LPS or vehicle treated adult and embryonic intestinal stromal cells, with the addition of control or CD14 siRNA knockdown in embryonic stromal cell cultures. N=3 biological replicates. t-test. *$p<0.05$

Ichinose et al., Suppl figure6

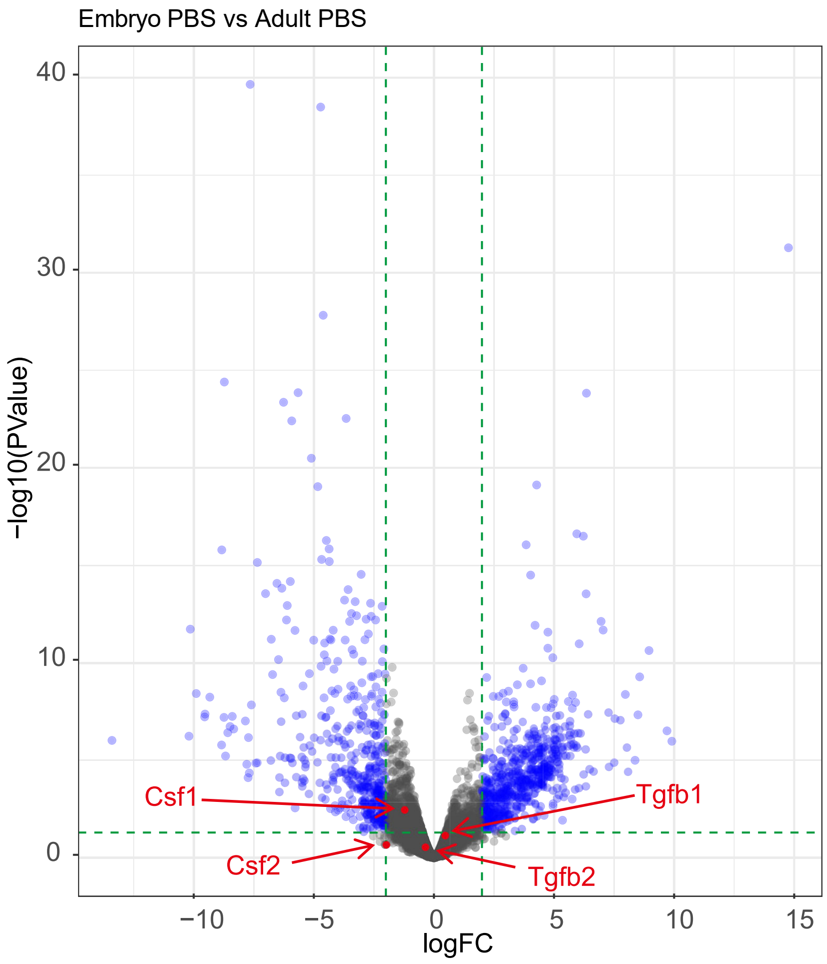

Suppl Fig.6 Volcano plot of differentially expressed transcripts between vehicle treated adult and embryonic intestinal stromal cells.

Colony-stimulating factor (CSF)-1,2 and transforming growth factor beta (TGFβ)-1,2 were not significantly differently expressed between embryonic and adult intestinal stromal cells. Absolute value of log2 fold change ≥ 2.0, FDR<0.01
